# Potential of AI-Driven Chatbots in Urology: Revolutionizing Patient Care Through Artificial Intelligence

**DOI:** 10.1007/s11934-023-01184-3

**Published:** 2023-09-19

**Authors:** Ali Talyshinskii, Nithesh Naik, B. M. Zeeshan Hameed, Patrick Juliebø-Jones, Bhaskar Kumar Somani

**Affiliations:** 1 Department of Urology, Astana Medical University, Astana, Kazakhstan; 2 Department of Mechanical and Industrial Engineering, Manipal Institute of Technology, Manipal Academy of Higher Education, Manipal, Karnataka 576104 India; 3 Department of Urology, Father Muller Medical College, Mangalore, Karnataka India; 4 Department of Urology, Haukeland University Hospital, Bergen, Norway; 5 Department of Clinical Medicine, University of Bergen, Bergen, Norway; 6 Department of Urology, University Hospital Southampton, Southampton, UK

**Keywords:** Chatbot, GPT, Artificial intelligence, Urology, Healthcare

## Abstract

**Purpose of Review:**

Artificial intelligence (AI) chatbots have emerged as a potential tool to transform urology by improving patient care and physician efficiency. With an emphasis on their potential advantages and drawbacks, this literature review offers a thorough assessment of the state of AI-driven chatbots in urology today.

**Recent Findings:**

The capacity of AI-driven chatbots in urology to give patients individualized and timely medical advice is one of its key advantages. Chatbots can help patients prioritize their symptoms and give advice on the best course of treatment. By automating administrative duties and offering clinical decision support, chatbots can also help healthcare providers. Before chatbots are widely used in urology, there are a few issues that need to be resolved. The precision of chatbot diagnoses and recommendations might be impacted by technical constraints like system errors and flaws. Additionally, issues regarding the security and privacy of patient data must be resolved, and chatbots must adhere to all applicable laws. Important issues that must be addressed include accuracy and dependability because any mistakes or inaccuracies could seriously harm patients. The final obstacle is resistance from patients and healthcare professionals who are hesitant to use new technology or who value in-person encounters.

**Summary:**

AI-driven chatbots have the potential to significantly improve urology care and efficiency. However, it is essential to thoroughly test and ensure the accuracy of chatbots, address privacy and security concerns, and design user-friendly chatbots that can integrate into existing workflows. By exploring various scenarios and examining the current literature, this review provides an analysis of the prospects and limitations of implementing chatbots in urology.

## Introduction

Currently, healthcare system is facing multiple burdens, related to demographic factors, social structure, and a lack of physicians [1, 2]. These problems have resulted in considerable impacts on healthcare globally, resulting in increased waiting times, compromised quality of care, and higher costs for patients [3]. Telemedicine services can shorten this gap by reducing medical visits, saving both the patient’s and the healthcare provider’s time and the cost of the treatment. Furthermore, due to its fast and advantageous characteristics, it can streamline the workflow of hospitals and clinics [4]. This disruptive technology would make it easier to monitor discharged patients and manage their recovery [5]. Chatbots are one of the forms of realization of such advantages and may have a beneficial role to play in health care to support, motivate, and coach patients as well as for streamlining organizational tasks [6]. Among them, chatbots based on artificial intelligence (AI) have shown the best potential in fulfilling a broad range of goals [7, 8].

Urology represents a rapidly growing field with the introduction of modern treatments and technologies [9]. By harnessing the potential of AI-enabled chatbots, urology can embrace digital transformation, optimize resource utilization, deliver patient-centric care, and overcome the challenges posed by different constraints [10]. Despite the apparent benefits, the use of such chatbots in urology has not yet been explored [11]. From another perspective, it is important to acknowledge that chatbots come with their own set of concerns and limitations, posing challenges for their safe and effective use in healthcare settings [12]. This review aims to combine current evidence on the use of AI-based chatbots in urology and to provide valuable insights for future research and implementation in this field.

## Material and Methods

### Search Strategy

In April 2023, the publication search was done in several databases, including the ACM Digital Library, CINAHL, IEEE Xplore, PubMed, Google Scholar, and https://clinicaltrials.gov/ with the use of the following terms: “AI,””Artificial intelligence,” “Chatbot,” “GPT,” “Decision aid,” “Support,” “Urology,” “Prostate,” “Bladder,” “Kidney,” “Ureter,” “Symptoms,” “Screening,” “Follow-up,” and “Treatment.” According to findings, we structured scenarios where chatbots were used in urology or were discussed to be useful. Moreover, Google Search (https://www.google.com/) was used to define dedicated sources on urological chatbots which are not published.

### Inclusion Criteria

Due to lack of data and comprehensive knowledge, we included original and development studies, ongoing trials, reviews, editorial comments, conference abstracts, and book chapters in the English language, without publication date restriction, to better highlight all potentials for the use of AI-chatbots in urology.

### Exclusion Criteria

Papers not in English language, description of chatbots not with AI-structure.

### Studies Process

Two reviewers (A.T. and N.N.) identified all sources. All studies that appeared to fit the inclusion criteria were included for full review. Each reviewer independently selected and structured studies. If there was disagreement or discrepancy, the senior author (B.K.S.) made the final decision.

### Data Extraction and Analysis

We reviewed studies and extracted information related to the scenarios where chatbots were implemented in urology. We also looked for any arguments or evidence supporting the use of chatbots in urological patients. We did not conduct a quantitative analysis of the papers but rather used them to inform our understanding of the different scenarios where chatbots were used and their potential utility in urological field.

## Results

**Fig. 1:**
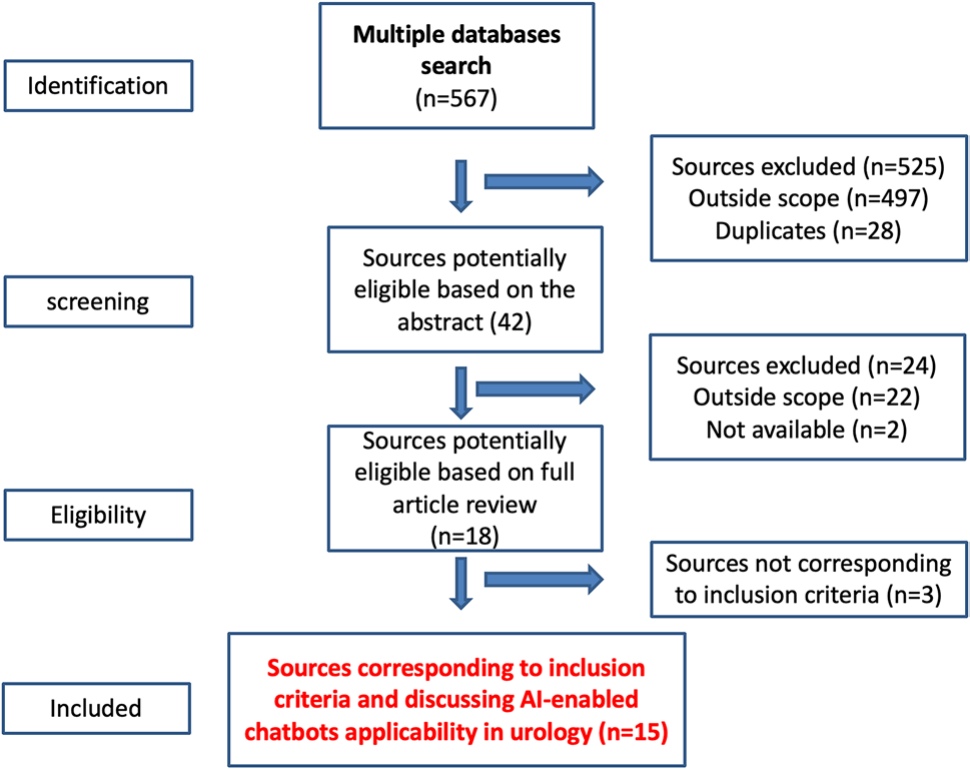
Flow diagram of included sources

Among 567 papers investigated (Fig. 1), 15 of these discussed different scenarios to use AI-based chatbots in urology. These included symptom checkers and health screening, patient education and counseling, lifestyle change and conservative management, and clinical decision support and post-treatment follow-up care (Table 1).

**Table 1:** Review of published urological literature on the use of chatbots

Authors	Type	Area	Function	Advantages	Limitations
Temsah et al. [10]	Review	General	Decision-making	Improves efficiency and reduced errors in decision-making	Accuracy of the generated output
Gabrielson et al. [11]	Editorial comment	general	Decision-making	ChatGPT has the potential by aiding in low-complexity tasks, facilitating the flow of information	May give wrong answers, cite non-existent publications, and provide meaningless answers due to algorithm bugs
Kobori et al. [13]	Develop and evaluation study	Sex-transmitted diseases	Screening	Total accuracy rate of a diagnosis of STI was 77.7%	n/a
Allen et al. [14]	Development and evaluation study	Prostate cancer	Education, Screening	Significant increase in prostate cancer knowledge and decrease in decisional conflict	n/a
Owens et al. [15]	Development and evaluation study	Prostate cancer	Education, screening	Participants experienced significant improvements in their prostate cancer knowledge and informed decision-making self-efficacy	n/a
Mark et al. [16]	Book chapter	Prostate cancer	Genetic counselling	Alternative delivery source for genetic counselling for men with prostate cancer	n/a
Huri and Hamid [17]	Editorial comment	Neurourology	Education, screening	Can keep the most vulnerable patients out of the hospital and help to fast-track patients that need to be seen for necessary investigations	Medicolegal issues, concerns about informed constant
Wang et al. [18]	Development and evaluation study	Sex-transmitted diseases	Education	Enables vulnerable and hard-to-reach population groups to talk and learn about sensitive and important issues	n/a
Görtz et al. [19]	Development and evaluation study	Prostate cancer	Patients education	Benefits patients as an additional informative tool	n/a
Khawam et al. [20]	Ongoing RCT	Urinary Incontinence	Education	n/a	n/a
Ray et al. [21]	Development study	Andrology	Education, mental health support	Physiological and psychological treatments for patients with erectile dysfunction, infertility, ejaculation problems, prostate gland issues	n/a
Kim et al. [22]	Develop and evaluation study	Interstitial cystitis/bladder pain syndrome (IC/BPS)	Patient education, behavioural modification, cognitive behavioural therapy, pelvic floor physical therapy	Useful in the self-management of IC/BPS symptoms with engagement ≥ 85% and accuracy ≥ 80%	n/a
Chen et al. [23]	Ongoing RCT	BPH	Self-management	n/a	n/a
Kim et al. [24]	Development and evaluation study	General	Decision-making	Stratify hospital patients according to medical filed (including urology)	Accuracy of the generated output
Goldenthal et al. [25]	Development and evaluation study	Urolithiasis	Follow-up	Alleviation of concerns surrounding common symptoms after ureteroscopy	Low patient engagement

### Symptom Checkers and Health Screening

Chatbots can be a useful tool in assessing symptoms associated with urological diseases. In the presence of mild or non-urgent symptoms, indications for referral to a specialist may be better defined. Conversely, complicated urological diseases can be life-threatening if missed, highlighting the importance of timely and affordable screening.

Kobori et al. [13] described the AI chatbot for sexually transmitted infections (STIs) screening, including gonorrheal and chlamydial urethritis, syphilis, and condyloma acuminatum. The accuracy rates of gonorrheal urethritis, chlamydial urethritis, syphilis, and condyloma acuminatum were 65%, 70%, 85%, and 95%, respectively. 97.7% of patients thought to visit to clinic earlier after they used a chatbot. Allen et al. [14] proposed a chatbot on decision-making for prostate cancer screening among African-American men, called Prostate Cancer Screening Preparation (PCSPrep). It significantly increased participants’ prostate cancer knowledge as well as reduced decisional conflict. Owens et al. [15] performed a paired study and found that iDecide decision-aid chatbots improved participants’ prostate cancer knowledge, self-efficacy in decision-making, and in the use of technology.

### Patient Education and Counseling

Chatbots can be used to educate and inform patients with urological diseases, helping to facilitate the routine practice of specialists [16]. They can ensure patients with information on urological conditions, treatment options, and preventive care measures, thus improving patient knowledge [17]. Wang et al. [18] developed SnehAI chatbot to handle with private topics such as safe sex and family planning. SnehAI provides a private and nonjudgmental space for users, offering reliable and relatable information and resources. The study utilizes the Gibson theory of affordances to examine SnehAI’s functionalities, and it demonstrates strong evidence across fifteen functional affordances, including accessibility, multimodality, compellability, interactivity, and inclusivity. PROSCA, developed by Görtz et al. [19], is a user-friendly medical chatbot specifically focused on prostate cancer (PC) communication. The study aimed to evaluate PROSCA’s effectiveness in providing patient information about early detection of PC. The chatbot proved to be straightforward to use, with a majority of users (78%) not requiring any assistance. Furthermore, 89% of the chatbot users experienced a clear to moderate increase in their knowledge about PC. The participants expressed their willingness to reuse a medical chatbot in the future, highlighting the support for chatbot integration in clinical routines. PROSCA demonstrated its potential in raising awareness, patient education, and support for early PC detection. Khawam et al. [20] are currently conducting a randomized controlled trial to investigate how usefully conversational ChatBot will provide education and help the patients with urinary incontinence.

### Lifestyle Change and Conservative Management

**Fig. 2:**
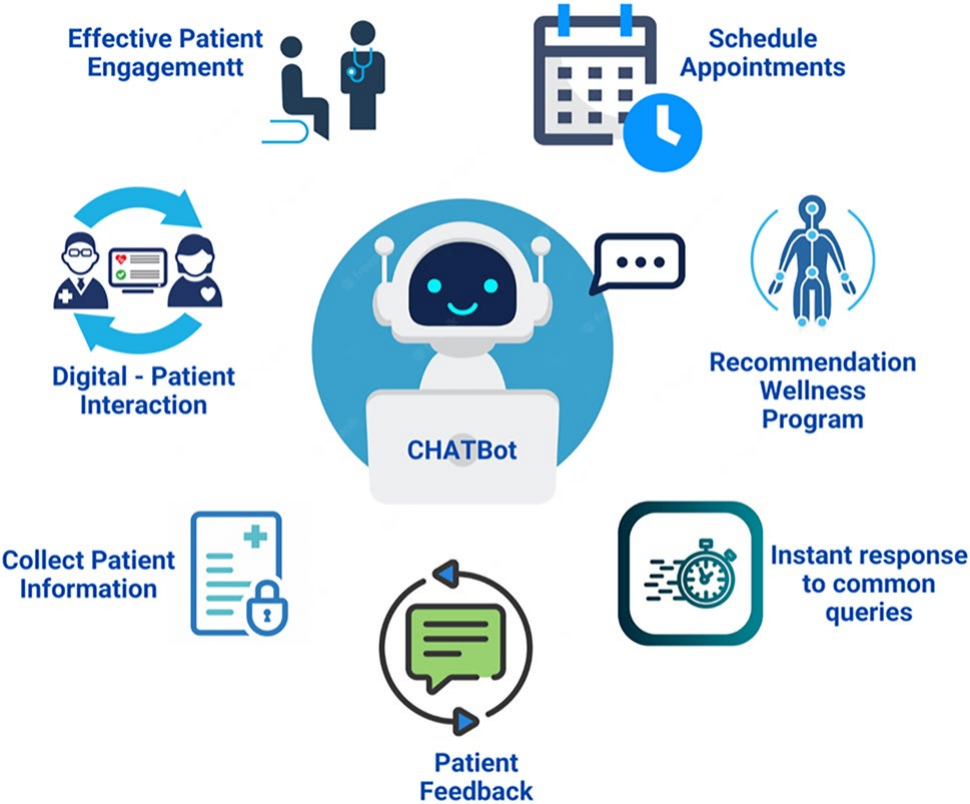
Examples of clinical applications of chatbot

AI-based chatbots hold significant promise for enhancing patient outcomes in urology, particularly for those patients who require adherence to treatment regimens and lifestyle modifications (Fig. 2). This is particularly relevant for a range of urological conditions such as urinary incontinence, urolithiasis, and erectile dysfunction, where making necessary changes to diet, exercise, and compliance to medications can significantly improve symptoms and quality of life. Ray et al. [21] introduced “MenGO,” an integrated cloud-based system for personalized andrological health management. MenGO addresses multiple systemic issues in the field of andrology and men’s sexual wellness by utilizing statistical modeling and a natural language processing (NLP) chatbot. This one-stop solution caters to men suffering from chronic ailments such as erectile dysfunction, infertility, ejaculation problems, and prostate gland issues. The system provides access to affordable physiological and psychological treatments through a smart and interactive telehealth platform powered by cloud and big data analytics. The NLP chatbot integrated into MenGO enhances the overall user experience by facilitating communication and reducing barriers in seeking appropriate healthcare. Kim et al. [22] developed a patient-centered text message-based platform to promote self-management of symptoms associated with interstitial cystitis/bladder pain syndrome (IC/BPS). The platform consisted of four treatment module categories, namely, patient education and behavioral modification, cognitive-behavioral therapy, pelvic floor physical therapy, and guided mindfulness practices. Supportive messages were delivered through an automated algorithm, enhancing the concept of provider support through shared decision-making and reducing the sense of isolation experienced by patients. This intervention empowered patients to manage their symptoms better, improve self-efficacy, and gain insight into their motivations and behaviors. Chen et al. [23] are currently conducting a randomized controlled trial to investigate how an AI chatbot intervention can impact the self-management and decision-making confidence of men with lower urinary tract symptoms (LUTS) caused by an enlarged prostate, with or without erectile dysfunction (ED), in the post-COVID-19 era. Patients have the opportunity to access the chatbot for free by scanning a QR code. The chatbot offers self-management guidance on topics including prostate enlargement, urinary symptoms, and erectile dysfunction. Moreover, it provides patient-centered decision-making tools aimed at supporting and empowering patients, particularly in relation to improving urination and erectile function.

### Clinical Decision Support

**Fig. 3:**
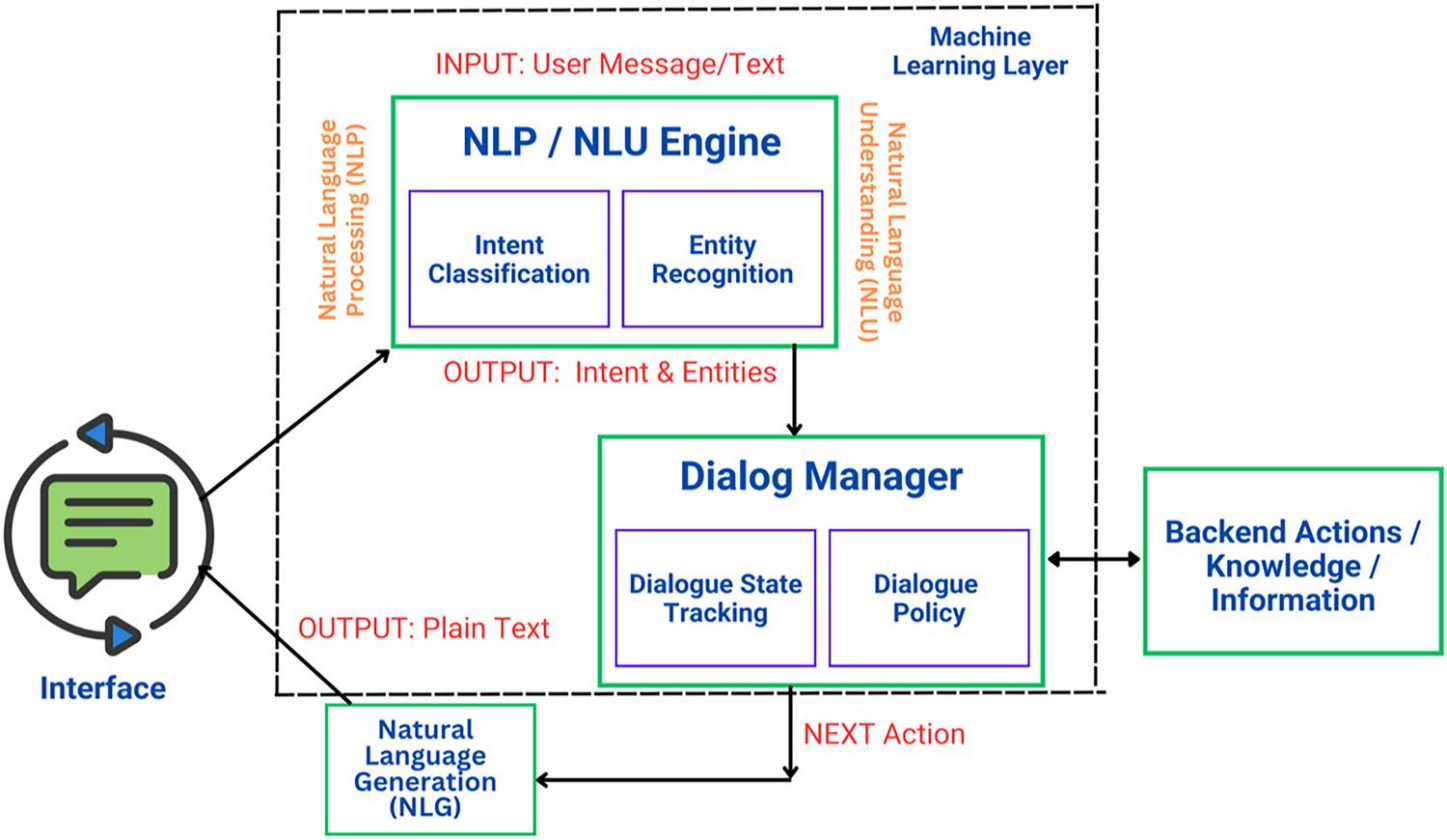
Example of chatbot mechanism of action

AI-based chatbots in urology can improve decision-making by analyzing patient data and medical records. By leveraging machine learning algorithms, these chatbots can provide evidence-based recommendations for diagnosis and treatment plans (Fig. 3). This assists healthcare professionals in interpreting complex urological data, leading to more accurate and efficient clinical decisions [10]. These AI-based chatbots can consider a wide range of patient variables and provide personalized recommendations based on the specific case at hand. As stated by Gabrielson et al. [11], they may serve as an essential tool in the urologist’s armamentarium to step away from the computer and turn the physician’s chair back toward the patient. Kim et al. [24] propose a medical specialty prediction model from patient-side medical question text based on pre-trained bidirectional encoder representations from transformers (BERT). The dataset comprised pairs of medical question texts and labeled specialties scraped from a website for the medical question-and-answer service. The model was fine-tuned for predicting the required medical specialty labels among 27 labels from medical question texts including urology. By analyzing the patient’s symptoms and complaints, chatbots can provide recommendations on which specialists in the field of urology or related areas should be consulted for a comprehensive examination and further diagnosis. This helps streamline the referral process and ensures that patients receive the most appropriate care based on their specific concerns.

### Post-treatment Follow-up Care

Chatbots can be highly useful in post-treatment follow-up in urology by providing continuous monitoring and support for patients. They can collect and analyze patient-reported outcomes, such as urinary symptoms or quality of life indicators, and provide personalized recommendations for self-care or additional interventions. As noted by Goldenthal et al. [25], chatbots could be programmed to follow up with patients after their urology appointments, checking in on their symptoms and providing guidance on self-care. This could be especially beneficial for patients who live far from their urology clinic or who have mobility issues that make in-person follow-up visits difficult.

## Discussion

Chatbots have been used in healthcare for decades, with the earliest known healthcare chatbot being ELIZA, created in 1966 [26]. Since then, chatbots have been utilized in various healthcare settings, from helping patients manage chronic conditions to providing mental health support. There are three main types of healthcare chatbots based on the input processing and response generation method: rule-based model, retrieval-based model, and generative model [8]. Rule-based chatbots use pre-programmed responses to provide information to patients, while retrieval-based bots offer more flexibility as it queries and analyzes available resources. The generative model produces chatbots based on machine or deep learning to improve their responses over time and is more promising for several reasons. They can understand natural language and learn from user interactions to provide more personalized responses. Additionally, AI-based chatbots can be used to analyze large amounts of patient data to identify patterns and trends that can be used to improve healthcare outcomes [27]. GPT (generative pre-trained transformer) is a well-known representative of such AI-chatbots and has been the subject of many discussions [28].

There are ample studies in the literature describing the potential prospects and applications of chatbots in urology. According to our findings, they are already in the focus of urological symptom checking, health screening, patient education, counseling, lifestyle change, conservative management, clinical decision support, and post-treatment follow-up care. The same was shown by Calvo et al. [29], who conducted a study on the feasibility and usability of a text-based conversational agent that processes a patient’s text responses and short voice recordings to calculate an estimate of their risk for an asthma exacerbation. The chatbot offers follow-up information for lowering risk and improving asthma control, to improve understanding and self-management of the condition. Ferré et al. [30] developed a chatbot-based tool, called the MyRISK score, which collects self-reported patient data before pre-anesthetic consultation to stratify patients according to their risk of postoperative complications. The tool was developed using the Delphi method and logistic regression analysis, with a machine learning model trained to predict the MyRISK score. The tool was found to be effective in predicting postoperative complications, with high sensitivity (94%) but low specificity (49%).

However, this is not a full list of their potential, which is obvious looking at papers investigating AI-chatbots in different medical fields. They can be also valuable for medical education, pre-operative preparation, and academic writing. So, Han et al. [31] developed an AI chatbot educational program to promote nursing skills and found that the experimental group showed significantly higher interest in education and self-directed learning compared to the control group. These studies collectively suggest that chatbots hold promise as a valuable tool for medical education. Chetlen et al. [32] went about the deployment of a chatbot to provide evidence-based answers to frequently asked questions for patients scheduled to undergo a breast biopsy procedure.

By streamlining processes and reducing wait times, chatbots can increase the overall efficiency of the healthcare system, leading to cost savings for healthcare organization [33]. They can also prevent unnecessary office visits and hospitalizations by providing patients with timely and accurate information and support. Chatbots can increase patient engagement and satisfaction by offering personalized advice, information about their condition, and treatment options [34]. Studies have shown that chatbots are more engaging and interactive than traditional online forms, despite taking longer to complete [35•]. According to systematic review from Geoghegan et al. [36], engagement rate for chatbots in the follow-up of patients who have undergone a physical healthcare intervention was up to 97%. In summary, chatbots have the potential to revolutionize the field of urology by improving patient care, optimizing workflow, and increasing the efficiency of the healthcare system.

### Technical Limitations

One of the major challenges facing chatbots in urology is their technical limitations. While chatbots have the potential to improve patient care and physician efficiency, they may not always function as intended due to technical issues. For example, chatbots may experience system failures, errors, or glitches that can affect their performance and accuracy [37]. This can be particularly concerning when it comes to providing medical advice or making diagnoses, as any errors or inaccuracies could lead to serious harm to patients. Therefore, it is essential to thoroughly test chatbots and ensure that they are operating correctly and providing accurate information.

### Privacy and Security Concerns

Another challenge that needs to be addressed when using chatbots in urology is privacy and security [38]. Chatbots may store personal health information, which raises concerns about data privacy and security. Patients need to be assured that their personal information is secure and protected from unauthorized access. Furthermore, any data breaches or security incidents could have significant consequences, including loss of patient trust and legal repercussions. Data privacy and security in health chatbots are still under-researched, and related information is underrepresented in scientific literature [39] Therefore, chatbots must comply with relevant privacy and security regulations to safeguard patient data.

### Reliability and Accuracy

Ensuring reliability and accuracy is one of the most crucial factors for the success of chatbots in urology. To provide trustworthy information to patients and healthcare providers, they must be developed with reliable data sources and algorithms [40••]. They should also undergo continuous testing and updates to maintain their reliability and accuracy over time. However, if chatbots are not reliable or accurate, they could misinform patients, leading to incorrect diagnoses or treatments. For instance, Ben-Shabat et al. [41] evaluated the data-gathering function of eight chatbot symptom-checkers and found that the overall recall rate for all symptom-checkers was 0.32 (2280/7112; 95% CI 0.31–0.33) for all pertinent findings. These results suggest that the data-gathering performance of currently available symptom checkers is questionable. As new chatbots become available, hypotheses about their future utility in medicine are limited only by researcher’s imagination. However, their current use should be limited to low-risk tasks with continued human oversight [11]. Regarding to scientific writing, several ethical issues arise about using these tools, such as the risk of plagiarism and inaccuracies, as well as a potential imbalance in its accessibility between high- and low-income countries, if the software becomes paying. For this reason, a consensus on how to regulate the use of chatbots in scientific writing will soon be required [42•].

### Resistance to New Technology

Chatbots in healthcare face the challenge of resistance from patients and healthcare providers who are unfamiliar with new technology or prefer face-to-face interactions. To overcome this challenge, chatbots need to be designed to be user-friendly and easily integrated into existing workflows. Healthcare providers should also receive training on how to effectively use and recommend chatbots. As Goldenthal et al. [25] indicated, frequent reasons for not activating the chatbot included misplacing instructions for chatbot use, relying on follow-up with clinic or discharge materials, inability to activate chatbot, and inability to text. Moreover, they are not capable of empathy, notably to recognize users’ emotional states and tailoring responses reflecting these emotions. The lack of empathy may therefore compromise the engagement with health chatbots [43].

### Limitations of Our Review

Our review has some limitations that need to be addressed. Firstly, we focused only on AI-based Chatbots, and incorporating other types of chatbots could expand their clinical application. Secondly, we conducted a qualitative assessment of the literature without collecting all identifiable articles, which could have provided a more comprehensive analysis. Thirdly, some scenarios for the use of AI bots could be further subdivided or combined. However, the purpose of our work was to provide an overview of the prospects and limitations of AI-chatbots in urology. By examining the current literature and exploring various use cases, we hoped to provide an analysis of the potential benefits and drawbacks of their implementing.

## Conclusion

The use of AI-driven chatbots in urology has the potential to revolutionize the discipline by enhancing patient care, raising physician productivity, and lowering healthcare costs. Chatbots can offer patients individualized assistance, information, and guidance throughout their healthcare journey, empowering patients to better manage their ailments and make educated decisions. Additionally, chatbots can help medical professionals by streamlining repetitive tasks like scheduling appointments, managing medications, and tracking symptoms, giving them more time to concentrate on challenging or complex patient cases. The implementation of chatbots in urology must, however, successfully navigate several obstacles. Obstacles include technical constraints, privacy and security worries, reliability and accuracy problems, and reluctance to new technologies. Chatbots must be created with trustworthy data sources and algorithms, rigorously tested, and frequently updated to maintain their performance and accuracy to accomplish this. To protect patient data, they must also adhere to pertinent privacy and security laws. Furthermore, chatbots must be created to be user-friendly and simple to incorporate into current healthcare workflows.

To employ chatbots to improve patient care, healthcare professionals and patients must receive the proper training and assistance. To provide patients with a more tailored and interesting experience, chatbots should also be able to demonstrate empathy and comprehend their emotional states. Overall, our exhaustive analysis reveals that AI-driven chatbots have the potential to revolutionize urology by enhancing patient care, raising physician productivity, and lowering medical expenses. To ensure their safe and successful application in clinical practice, they must first be carefully evaluated in light of their difficulties, limitations, and ethical considerations.

## Data Availability

Data is available on request.
